# Concordance between Preoperative #ENZIANi Score and Postoperative #ENZIANs Score Classification—Why Do We Choose #ENZIAN and How Does It Impact the Future Classification Trend?

**DOI:** 10.3390/jcm13196005

**Published:** 2024-10-09

**Authors:** Zofia Borowiec, Maja Mrugała, Krzysztof Nowak, Wiktor Bek, Ewa Milnerowicz-Nabzdyk

**Affiliations:** The Clinical Department of Oncological Gynecology, Oncology Centre in Opole, Medical Faculty, University of Opole, 45-061 Opole, Poland; maja.mrugala@gmail.com (M.M.); knowakmd@gmail.com (K.N.); wiktor.bek@gmail.com (W.B.); ewa.milnerowicz@wp.pl (E.M.-N.)

**Keywords:** #ENZIAN, minimally invasive surgery, endometriosis classification system, deep endometriosis

## Abstract

**Objectives:** To assess the concordance of the preoperative application of the #ENZIAN classification (#ENZIANi) with the postoperative result (#ENZIANs) using surgical findings as the reference standard. **Methods:** This retrospective study included 282 consecutive patients with deep endometriosis undergoing surgical treatment. Preoperative assessment with transvaginal sonography and magnetic resonance imaging was compared with postoperative assessment. Concordance and diagnostic test evaluation were calculated. **Results:** The highest concordance was observed in the F (abdominal wall endometriosis) with k Cohen of 0.837, following the values for pelvic locations, with 0.795 for T left, 0.791 for T right, 0.776 for F (adenomyosis), 0.766 for C (rectum), and 0.75 and 0.72 for O right k and O left, respectively. The highest sensitivity was demonstrated for the P compartment *(98%), T compartment (both sides 97%), and A, B, C (94–96%), corresponding with deep endometriosis. **Conclusions:** Preoperative assessment using TVS/TAS + MRI with the ENZIANi score correlates well with the ENZIANs postoperative score and demonstrates good concordance in the detection and localization of deep endometriosis, thereby minimizing false negative results and ensuring accurate preoperative staging. The ENZIAN classification is well-suited to surgeon needs and benefits from continuous development. Future improvements, such as adding the expanded C module, may be considered in the next edition.

## 1. Introduction

Endometriosis affects 1 in every 10 women today, as reported by published data [1,2]. Recognizing the growing interest in this complex pathology, which profoundly impacts women’s health, fertility, and healthcare systems worldwide, the search for the newest and most effective systems of professional assessment of this disease is of the highest importance.

Endometriosis surgery has become one of the most complex and advanced domains of contemporary surgery, presenting challenges that affect the entire team of the gynecology ward and the operating theatre. Consequently, the need for precise diagnosis and predictive planning is crucial for effective and safe surgery [3].

The safety of the patient and surgeon, adequately planned time and equipment in the operating theatre, and the advantage of discussing a comprehensive and adapted consent form with the patient before the operation are among the most valuable issues in a surgeon’s work today.

The diagnostic system for deep endometriosis, #ENZIAN, has been interesting since its inception [4]. This intuitive and anatomical classification, created by Professor J. Keckstein, is tailored for endometriosis surgeons. In our opinion, it enhances the surgical approach and improves communication between radiologists and gynecologists, as well as interpersonal communication on an international level, which is why it has become useful in everyday practice in our Centre. It enhances the surgical approach and improves communication between radiologists and gynecologists, as well as interpersonal communication on an international level. Therefore, the aim of our study was to assess the concordance of the preoperative application of the #ENZIAN classification (#ENZIANi) with the postoperative result (#ENZIANs) using surgical findings as reference standard in a group of complex endometriosis cases surgically treated in Centre of Oncology in Opole, Poland. 

## 2. Materials and Methods

The project received approval from the bioethics committee Medical Faculty, University of Opole reference number UO/0020/KB/2022, and was conducted in line with its assumptions. This report conforms to the Strengthening the Reporting of Observational Studies in Epidemiology (STROBE) guideline for observational studies [5].

Using the #ENZIAN classification system at our specialized center, the Oncological Gynecology Department at the Oncology Centre in Opole, Poland, we have been analyzing the preoperative staging of endometriosis and postoperative reassessment. This approach aims to enhance our ability to accurately predict the type and duration of procedures required for each patient.

### 2.1. Population

The database of the Oncological Gynecology Clinical Department at the Oncology Centre in Opole, Poland, was retrospectively searched from 1 January 2020 to 31 December 2023.

Exclusion criteria included a history of co-existing cancer, post-menopausal status, and incomplete preoperative exam data (MRI, ultrasound, detailed medical and surgical history). The ward does not admit juvenile, pregnant, or male patients. We included patients who underwent surgery for deep endometriosis and were prepared with an adapted preoperative protocol, with mandatory expert ultrasound and MRI diagnosis. Since the new #ENZIAN classification was published in 2021, retrospective reassessment was conducted for patients in 2020 and 2021, as we began using #ENZIAN in 2022. 

Overall, 282 consecutive patients with deep endometriosis were involved in this study. These findings are intended to improve our surgical methods, enhance patient safety, and increase the effectiveness of endometriosis treatment, ultimately leading to better surgical planning and detailed explanation of the procedure to the patient. 

### 2.2. Ultrasound Examination

**Figure 1:**
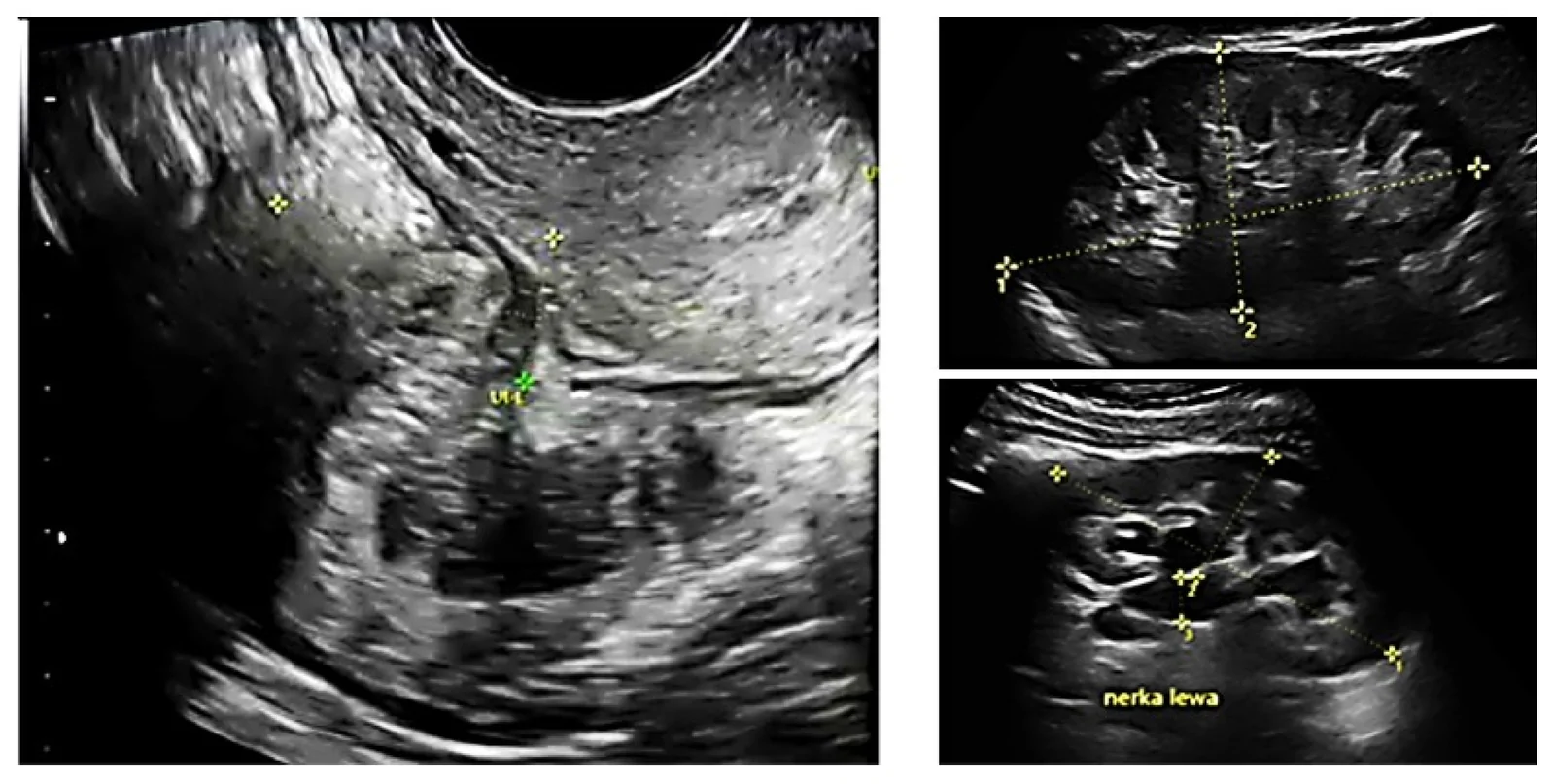
Preoperative ultrasound examination: the bowel DE nodule (TVS exam), congestion in the left kidney (TAS exam).

All patients underwent preoperative ultrasound examination by an expert surgeon-gynecologist—(E.M.-N.), with detailed pelvic compartments assessment via transvaginal sonography (TVS) and with kidney congestion assessment via transabdominal sonography (TAS), following an adapted protocol-based on IDEA group guidelines [6]. Examinations were performed on Voluson E8 Expert with the use of vaginal probe RIC5-9-D (depth max 16 cm) and abdominal probe RAB2-5-D, mainly for the kidney scan. A detailed report was recorded for each patient. Examples of ultrasound examination images are shown in Figure 1.

### 2.3. The Pelvic MRI Scan

The project assumed that each included patient would undergo an MRI examination at a specialized MRI facility collaborating with the Gynecological Oncology Department. As an exception, 11 scans performed at other MRI facilities were accepted, considering the risk of decreasing the accuracy of the description, setting a condition that the examinations would be conducted according to similar protocols. The current preparation protocol for MRI examinations and the technical parameters were consistent with ESUR guidelines [7].

**Figure 2:**
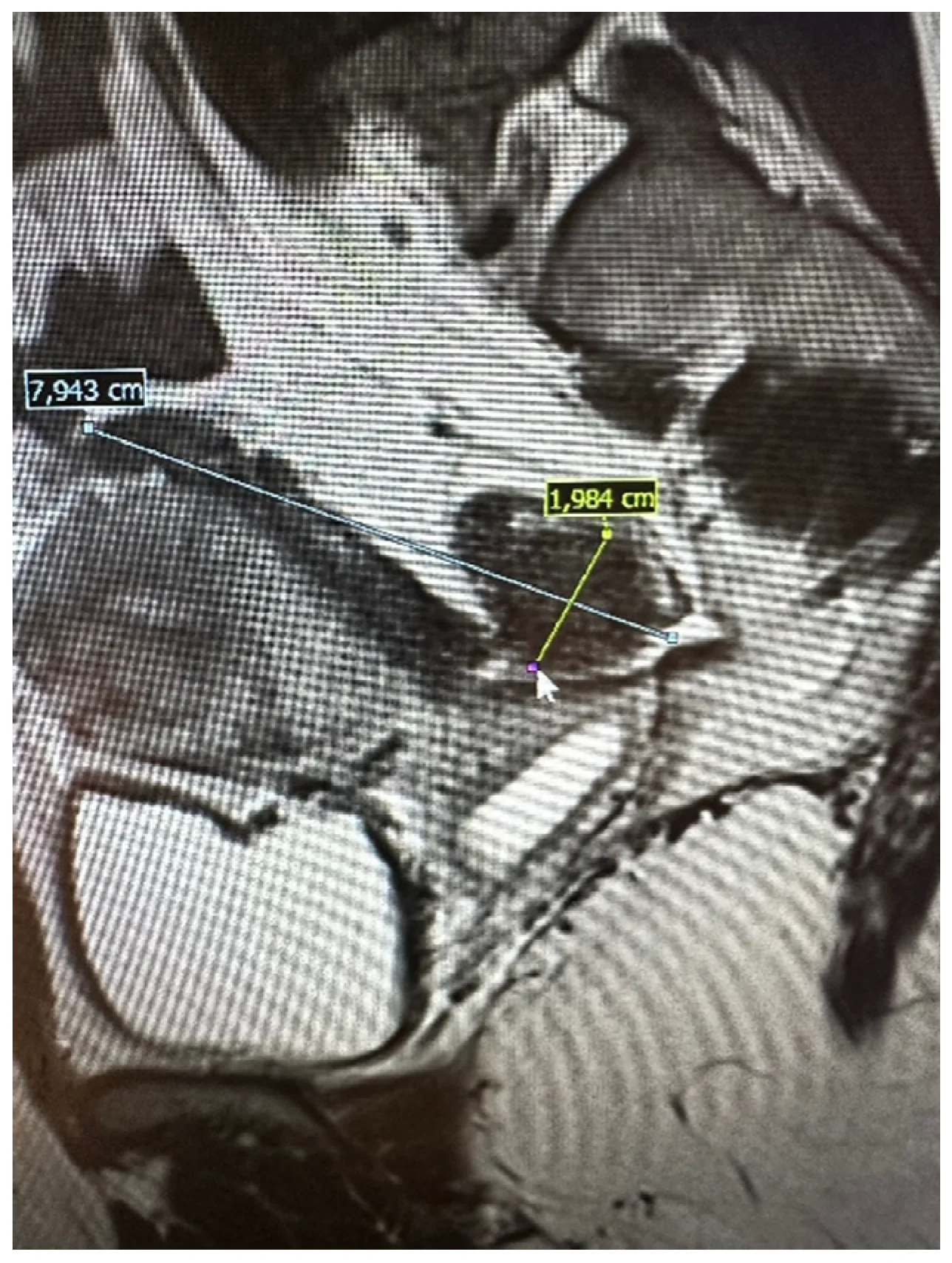
Preoperative magnetic resonance examination. The same bowel DE nodule as presented in TVS above (compare Figure 1).

The pelvic MRI was conducted using a 3T scanner, following the established protocol for endometriosis. This included scans before and after the intravenous administration of a gadolinium-based contrast agent, as well as contrast administration into the vagina (using hyaluronic acid gel and a vaginal tampon) and rectum (using a water solution). Patients were positioned supine with a moderately filled bladder and received an intravenous anti-peristaltic agent (butyl-scopolamine). The procedure was performed following bowel preparation (BPP) and fasting the previous day. The examination typically lasted approximately 60 min. The report included the abdominal wall structures. An example of an MRI scan is shown in Figure 2.

### 2.4. Other Imaging Techniques 

In individual cases of diaphragmatic, pulmonary, or upper abdominal endometriosis, additional diagnostic tests specific to the affected organ/area were performed. The chest was examined using computed tomography (CT) [8], as this is the first-line diagnostic modality for bronchopulmonary and pleuropulmonary endometriosis. The diaphragm and upper abdominal organs were examined with targeted magnetic resonance imaging (MRI) of the abdomen, including the diaphragm, as the modality of choice [9,10,11].

### 2.5. Surgery 

The surgical procedures were conducted at the ESGO-accredited Training & Surgical Centre, one of the few hospitals in Poland performing the most complex surgeries and handling the highest number of deep endometriosis cases per year. The lead surgeon for all procedures was (E.M.-N.) assisted by her specialized team. Surgery was tailored to each patient, respecting their needs and following the scheme created with the compilation of modern and safe minimally invasive techniques with the use of ICG was the approach of choice. The support of a multidisciplinary team was available if needed (thoracic surgeon, urologist, oncology surgeon, neurosurgeon). The guidelines of the working group of the European Society for Gynecological Endoscopy (ESGE), European Society for Human Reproduction and Embryology (ESHRE), and the World Endometriosis Society (WES) recommendations on the practical aspects of surgery for the treatment of deep endometriosis [12] and surgical safety measures were adequately respected.

**Figure 3:**
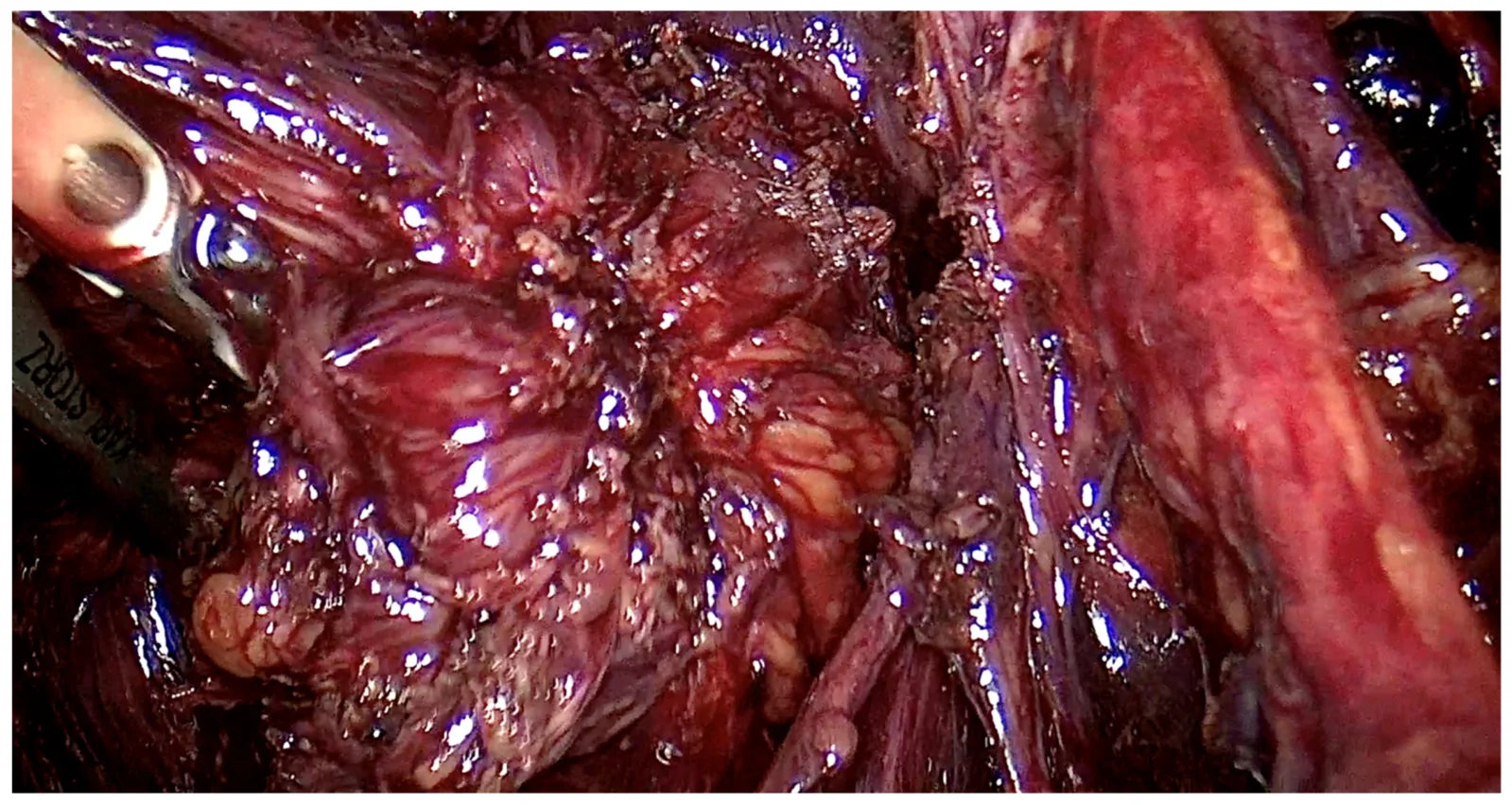
Intraoperative image of laparoscopic procedure—deep endometriosis affecting the bowel the nodule before the excision.

**Figure 4:**
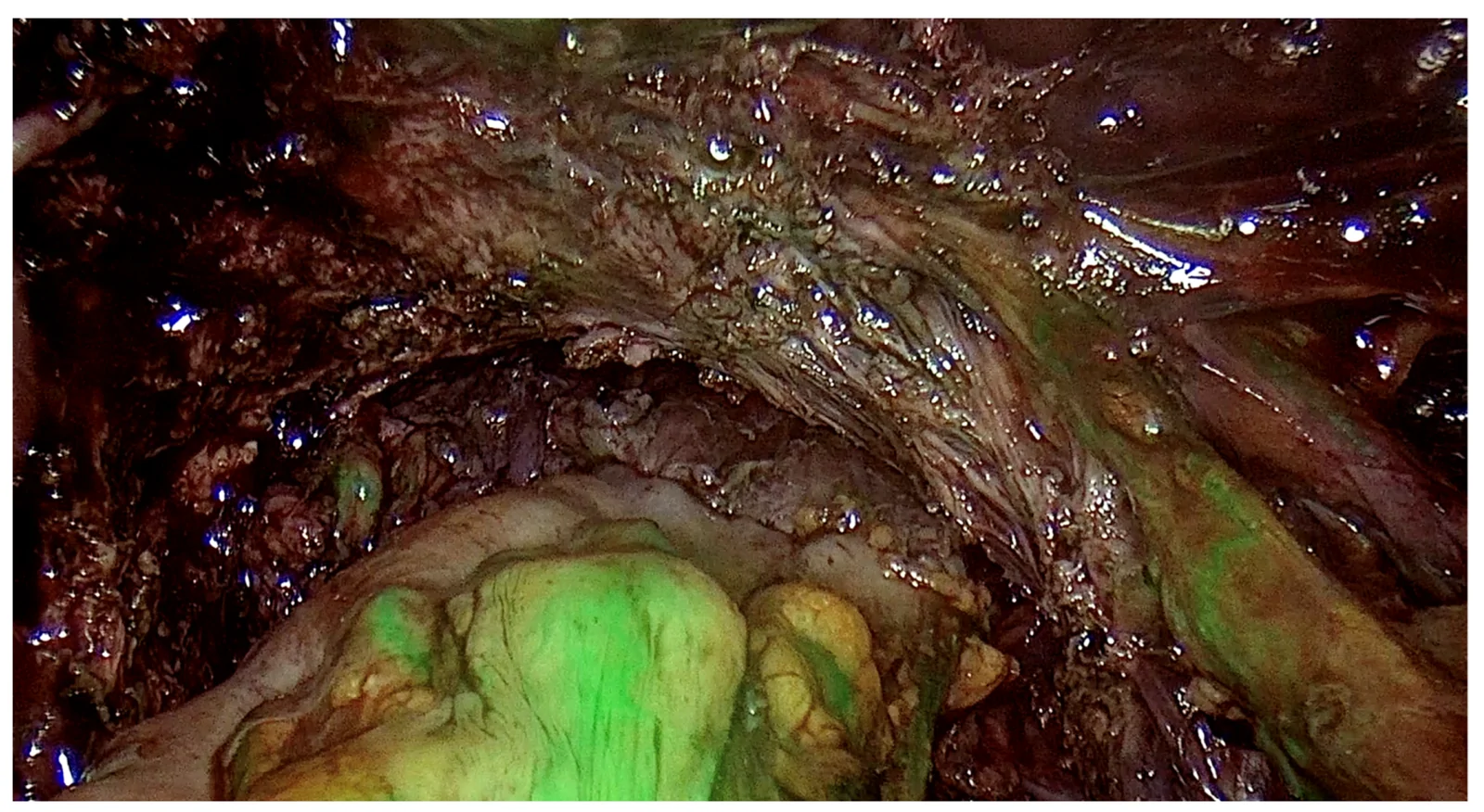
Intraoperative image of laparoscopic procedure—the ICG testing of the bowel after the nodule excision.

The range of the surgery was wide and consisted of modified radical hysterectomy, unilateral or bilateral infiltration of USLs and RVS resection, unilateral or bilateral ovarian cystectomy or drainage, large bowel resection procedures (shaving, discoid resection, segmental resection of the rectum, sigmoid colon, caecum), partial bladder resection, unilateral or bilateral ureterolysis sometimes with ureteral retransplantation, small intestine segmental resection, adnexectomy, nephrectomy, diaphragmatic lesions resection, bone, nerve and vessel lesion resection, and 2 cases of thoracic interventions. An image of the laparoscopic procedure with advanced bowel surgery is shown in Figure 3 and Figure 4.

### 2.6. Study Design

Given the personal experience and the availability of both imaging methods (TVS and MRI) for each patient qualified for surgery, the following sequence for assessing disease progression was decided upon.

Patients were evaluated on the #ENZIAN scale using two main criteria:

1. Preoperative assessment (#ENZIANi = imaging, TVS + MRI):

This assessment combined the findings from ultrasonography performed by the first surgeon qualifying and performing the surgery at each of these patients (E.M.-N.), using both vaginal and transabdominal probes, with the changes described in MRI reports by a radiology expert, using a 3T machine.

2. Postoperative assessment (#ENZIANs = surgery):

This assessment evaluated the changes observed during surgery.

The choice of combined imaging (TVS + MRI) facilitated the compilation of results and the preparation of a single, summed #ENZIANi scale before surgery. This approach combined the advantages of both imaging methods, allowing for quicker conclusions regarding disease progression and improved communication within the team. 

### 2.7. Statistical Analysis

Data were collected in an Excel spreadsheet (Microsoft Excel 2013; Microsoft Corp., Redmond, WA, USA) and statistically analyzed using MedCalc v. 19.5.3 (MedCalc Soft-ware Ltd., Ostend, Belgium). Data were presented as means with standard deviation, medians, ranges, numbers, and percentages depending on the type of data distribution. The concordance between preoperative TVS, TAS, and MRI examinations and surgical assessments for the involvement of #ENZIAN compartments was evaluated using Cohen’s kappa. This coefficient refers to measures of the agreement between two raters or measurements. Cohen’s kappa is used to evaluate how well two or more judges agree on their assessments or classifications, adjusting for agreement that could happen by chance. Interpretation of Cohen’s kappa values: a value was interpreted following Altman’s recommendations as poor (<0.20), fair (0.21–0.40), moderate (0.41–0.60), good (0.61–0.80), and very good (0.81–1.00) [13]. To graphically present concordance, correlation heatmaps were used. In addition, a diagnostic test evaluation was conducted. The sensitivity and specificity of TVS, TAS, and MRI for detecting endometriotic lesions/adhesions in each #ENZIAN compartment were calculated along with the positive predictive value (PPV), negative predictive value (NPV), and accuracy of TVS and TAS in identifying endometriotic lesions/adhesions in the various #ENZIAN compartments. The results of diagnostic test evaluation were presented as percentages with 95% confidence intervals (CIs) [14]. For the diagnostic test evaluation, the following assumptions were made: true negative was defined when both were negative, true positive was defined when both results were above 0, the false negative was defined when imaging was negative, and surgery indicated a lesion of 1–3, and false positive was defined when imaging detected a lesion of 1–3, and surgery was negative. Cases with m, x, and missing data were excluded.

## 3. Results

### 3.1. Population 

Out of the 307 women operated on for deep endometriosis (DE) in the Gynecological Oncology Ward at the Oncology Centre in Opole, Poland, between 1 May 2020 and 31 December 2023, according to the criteria, 24 were excluded from the final cohort. The group meeting the inclusion criteria comprised 282 women. The mean age of women was 39.22 ± 5.78 years. 

### 3.2. Concordance 

The statistical analysis was aimed to assess the level of concordance between the imaging techniques, consisting of the expert TVS/TAS and expert MRI, assessed together as #ENZIANi and intraoperative classification result, described as #ENZIANs. 

The K value was interpreted following Altman’s recommendations as poor (<0.20), fair (0.21–0.40), moderate (0.41–0.60), good (0.61–0.80), and very good (0.81–1.00) (Altman 1990).

Amongst the pelvic locations, the highest concordance with the highest k Cohen factor was observed in the tubo-ovarian area T left k Cohen 0.795 and T right k Cohen 0.791, in Fa (adenomyosis) k Cohen 0.776; C (rectum) k Cohen 0.766; and both ovaries O right k Cohen 0.75 and O left k Cohen 0.72.

The slightly lower results were achieved in the A, B, and Fi intestines, respectively. Compartment A (vagina, retrovaginal space) had a k Cohen coefficient of 0.69. In compartment B (sacrouterine ligaments, cardinal ligaments, pelvic sidewall), for B right, k Cohen was 0.653, and for B left, k Cohen was 0.643. For the intestines (16 cm above the Z line), the k Cohen coefficient equaled 0.647. The lowest concordance was observed for the peritoneum P with k Cohen of 0.519, alongside the F bladder k Cohen 0.448; F ureter, P nerve, F muscle, F bone, F lung, as represented in the table below.

Values x, m, and cases where measurements were missing were excluded from the calculations. 

The highest concordance was observed in the abdominal wall endometriosis—additionally assessed in this study—k Cohen 0.837 as those locations were diagnosed with special attention to the indicated location, often with additional measures. 

**Figure 5:**
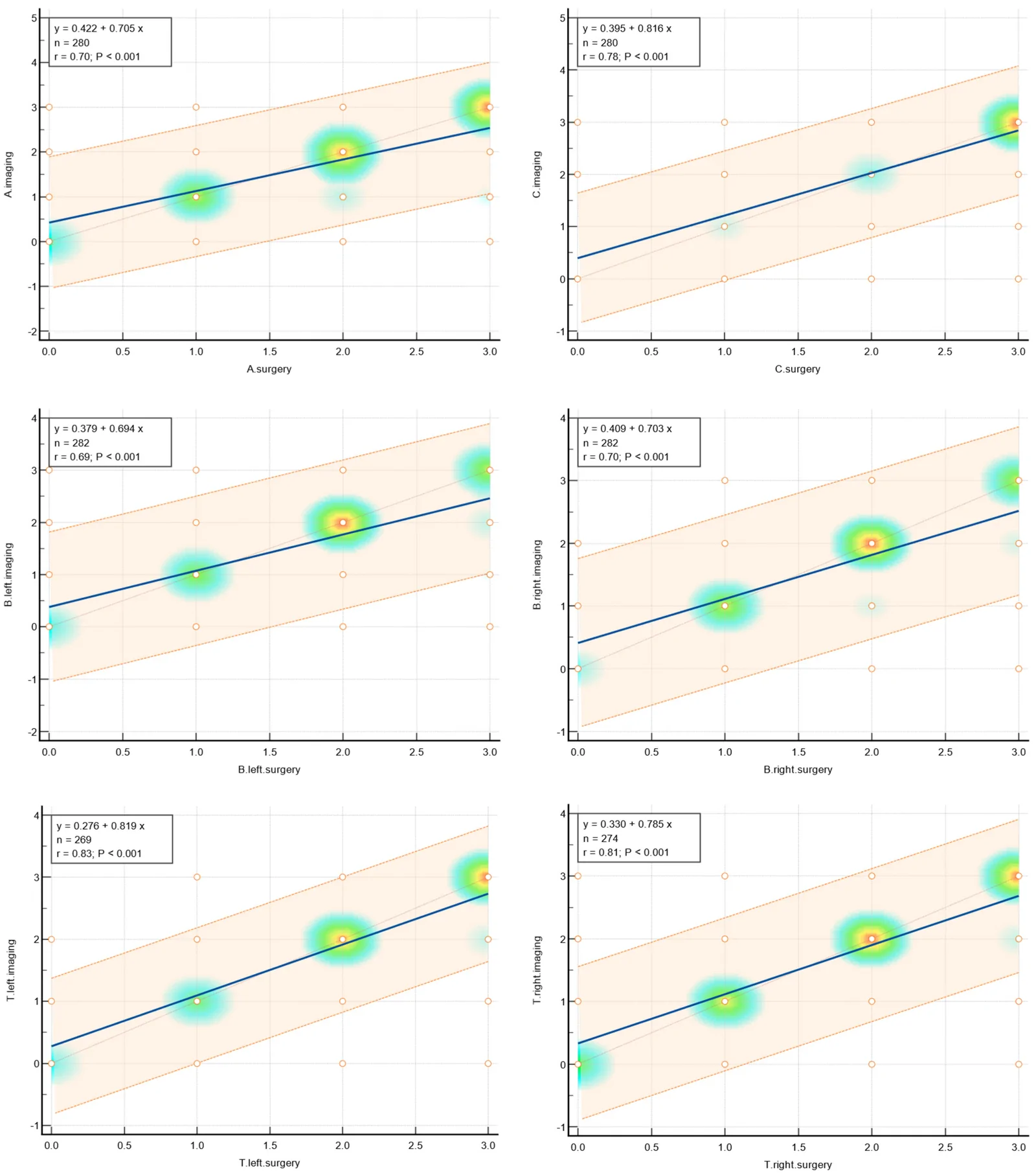
Heatmaps showing agreement for selected parameters.

The values of concordance are shown in Table 1. Graphically, concordance was illustrated by correlation heatmaps depicted in Figure 5.

### 3.3. Diagnostic Test Evaluation

The highest sensitivity was presented for the P and T compartments, and the following A, B, and C corresponded with deep endometriosis. Diagnostic test evaluation is shown in Table 2.

## 4. Discussion 

The matter of effective deep endometriosis imaging has been intensively analyzed in gynecological publications lately. As the multiple specialized centers started introducing their own protocols, the number of comparable studies is consistently growing, and the guidelines of working groups are being published, the future seems to be promising.

As for now, the #ENZIAN classification seems to be of the most frequent use, and its value has been confirmed in many trials, attributing not only to the education of surgeons, radiologists, and researchers but also to introducing new, wider than before, methods of operation planning [15].

According to the newest publication of the International Consensus published in May 2024 [16], the golden standard in the diagnosis of deep endometriosis is the expert TVS/TAS performed by the surgeon supplemented by an expert MRI, which is consistent with our approach of choice, presented in this work [17]. This has also been widely analyzed throughout the years and has recently been tested in a fusion exam mode [18].

To the best of our knowledge, this study represents the largest retrospective investigation into the preoperative use of the #ENZIAN classification for endometriosis based in one center, performed by the same assessing and operating team: one surgeon performing TVS/TAS (E.M.-N.), the same expert as the leading surgeon (E.M.-N.), one expert radiologist (except for 11 cases, as mentioned above), and one gynecologist specializing in endometriosis (Z.B.) collecting the data and scoring #ENZIAN for each patient [9].

The MRI-based ENZIAN score correlates adequately enough with the intraoperative findings, enabling better planning of the surgical procedure for patients and physicians [19].

Amongst the pelvic locations, the highest concordance with the highest k Cohen factor was observed in Fa (adenomyosis), C (rectum), T (tubo-ovarian), and O (ovaries).

In Di Paola’s retrospective study, which involved 82 women with deep endometriosis, MRI-based ENZIAN coding demonstrated good accuracy in detecting lesions across the various ENZIAN compartments: 81% for compartment A, 89% for compartment B, 82% for compartment C, 100% for compartment FA, and 37% for compartment FB [20], which results correspond very well with presented in this study. 

We are also aware of the time factor—the period of time between the ultrasound assessment, the MRI exam, and the operation. Even though the aim was to perform the above procedures shortly after each other, the service availability, patients’ personal reasons, the COVID-19 pandemic, and other arbitrary situations inevitably may have influenced the final results in some patients. The lesions observed in imaging could obviously evolve due to time, pharmacological treatment, and other diseases by the day of surgical intervention. We consider, though, that this is a universal condition that occurs in each specialized center. 

### 4.1. The B Compartment

The literature reports the poorest diagnostic performance for compartment B among all the individual parts of the #ENZIAN classification [21]. This finding of the study by Burla et al. are aligned. This was also a retrospective analysis including 63 patients.

Sensitivities and NPVs for compartments A, B, C, FA, FB, and FI were 95.2% and 91.7%, 78.4% and 56%, 91.4% and 89.7%, 57.1% and 94.1%, 85.7% and 98.3%, and 73.3% and 92.2%, respectively. The authors concluded that the #ENZIAN classification can act as an anatomical reference and enhance dialog between specialists, including radiologists and gynecologists [19]. Another valuable study was a retrospective MRI-based prediction analysis conducted by Thomassin-Naggara et al., which considered 150 women [3]. This study demonstrated concordance with the surgical #ENZIAN coding of 78.7% for compartment A, 34.7% for compartment B, and 82.7% for compartment C. Consistent with our findings, MRI showed the poorest diagnostic performance in compartment B. Accurate preoperative assessment should help surgeons anticipate challenging complications and the potential need for preoperative hormonal treatment, such as a gonadotropin-releasing hormone analog. Predicting the occurrence of de novo voiding dysfunction is of major importance. Although rated as a Clavien–Dindo grade II complication, it is one of the most dreaded complications; it requires self-catheterization and is a major determinant of postoperative alteration of quality of life with a risk of definitive sequelae in up to 3% of patients [3,22,23]. In our study, the B compartment was of moderate concordance, which in our opinion, owes to our experienced radiology center, assessing MRI exams. It was with huge enthusiasm that we greeted the #ENZIAN classification [14,24] after many years of inconvenience and communication problems when trying to describe the disease to the other physicians or to the patient, as rASRM grade IV was the only way to express the severity of the disease. For now, when we are fluent with the #ENZIAN and as many tertiary centers, we use it as our everyday work syllabus, we have a modest proposition of postulating some new development suggestions to the main scheme. As we are highly aware of the complexity of the #ENZIAN score, which has been noted by several authors (in comparison to the rASRM score, for example), we predict that any new addition to the main, clear, and concise core (POT ABC) of the classification may diminish its practical value. Therefore, we propose adding an optional extension to “the #ENZIAN app”, which would be available facultatively after entering the main data. This extension would cover advanced bowel surgery, abdominal wall surgery, and potentially detailed descriptions of the ureters. Future development of the #ENZIAN, as it is clear that the classification is ameliorating with the improvement of surgery, is warmly awaited.

### 4.2. Rectum (C)

Considering that the concordance in the C parameter is relatively high, one may say that the result of this predictive factor is satisfying. Nevertheless, regarding the capacity of an expert surgeon or radiologist for precise rectum examination, we would have the need to express it properly in the pure C parameter.

As an illustration, the same C3 score is given for a 3 cm flat infiltration as well as for, for example, three large full-thickness lesions of 3 cm each, which narrow the intestine, significantly changing the scope of the procedure.

Therefore, in our opinion, the C parameter would need to be expanded with some additional information to define precisely the scope of the disease: 1. number of tumors, 2. narrowing of the bowel lumen, 3. the depth of the bowel wall infiltration according to the layer, and 4. the distance from the anal verge seems to be necessary. Hence, if possible, “the #ENZIAN app” would be a field to be modified with this additional module, which could be facultative, added for tertiary care with intestinal surgery departments.

### 4.3. Location in Abdominal Wall (Fw) 

The #ENZIAN does not specify the parameter: abdominal wall endometriosis, where the lesions may also be extensive and may influence the scope of surgery and treatment planning. The parameter Fw can evidently be described in the F subgroup, but as it is much more prevalent than in other locations, its separate description may be worth attention. 

### 4.4. Ureters’ Assessment (Fu)

In our center’s experience, MRI poorly detects enlarged ureters—changes are most often described when significant ureteral enlargement is visible, often associated with kidney sclerosis. This is probably due to the ureter’s anatomy, as it becomes distended over the pelvic brim, which is located over the area covered by the pelvic MRI. “The diagnosis of ureteral endometriosis entails a high risk of renal disorder.” Imaging techniques offer limited value in accurately depicting the extent of ureteral lesions, as shown in Table 3. The diagnosis remains challenging, with surgical findings often differing from pre-surgical evaluations. This unpredictability requires the surgeon to be prepared for a range of possibilities, from encountering normal ureter anatomy to the need for JJ stenting or even ureteral transplantation. Preliminary results suggest that magnetic resonance urography is accurate in differentiating between intrinsic and extrinsic forms of ureteral involvement, but further studies are required to define its role in directing better treatment [25]. Taking into account the scientific publications over the last few years, the approach seems to be similar. The first argument for a routine renal assessment during an endometriosis scan is that “as many as 50% of patients with ureteral endometriosis are asymptomatic”. It is, therefore, difficult to obtain a prompt diagnosis, and ureteral endometriosis can lead to a subtle loss of renal function [26]. “Delayed diagnosis of ureteral endometriosis can lead to significantly decreased kidney function or even loss of obstruction of ureteral and hydroureteronephrosis” [27,28]. “Additionally, ureteral hydronephrosis should first be evaluated with renal ultrasound” [26]. “Renal ultrasound is the ideal first-line examination for ureteral endometriosis. In fact, it is most useful in detecting hydronephrosis, and it is non-invasive, reproducible, and cost-effective. Once the diagnosis is confirmed or suspected by means of renal ultrasound, several tools are available to confirm this diagnosis” [27]. Ureteral endometriosis is inevitably accompanied by endometriosis in the pelvic wall or the parametrium. Therefore, all patients who have deep endometriosis should be offered a renal ultrasound every six months to rule out urinary obstruction, which is an absolute indication for treatment. In the case of hydronephrosis, a urological diagnostic confirmation of renal function should be carried out preoperatively (retention parameters, renal scintigraphy, cysto-/ureteroscopy, MRI urography, excretory urography) [10]. “However, imaging techniques are of limited value in providing an accurate depiction of the extent of the disease and the infiltration of the ureteral wall. Abdominal ultrasound is routinely used as a screening tool to rule out urinary tract obstruction in patients with pelvic endometriosis, because of the high rate of silent presentations. The exam is simple and non-invasive, the evaluation requires no intravenous contrast and the findings are highly sensitive for hydronephrosis. Therefore, in clinical practice, periodic kidney ultrasound (every 6 months at first) from the time of diagnosis is generally suggested” [29]. Magnetic resonance imaging (MRI) may reveal direct signs such as a nodule or a mass invading the ureter along its course or at the ureterovesical junction. Nevertheless, the ureter is a hollow organ only 4–5 mm in diameter, and sometimes, it is difficult to analyze it with MRI as its spatial resolution reaches its limit [29]. “The most reliable assessment of ureteral involvement is achieved through examining kidney congestion via transabdominal ultrasound (TAS)” [14]. That is why, in our opinion, the ureteral assessment with the obligatory renal assessment could be another option in #ENZIAN App development.

### 4.5. Why Would a Surgeon Use a Score?

This last question precisely highlights the core issue: how to develop an ideal scoring system that would enhance communication, shorten the patient’s path to treatment, and address international variations in MRI descriptions and treatment options.

The main diagnostic challenge in dealing with endometriosis lies in its enormous variability and the polymorphic forms it presents from patient to patient. It can manifest as a delicate local lining of the peritoneum, causing severe pain, or as a deeply infiltrating bowel tumor, narrowing the lumen by 90% and causing no pain during examination, yet posing a significant risk of ileus.

Another significant issue is the interobserver variability in assessing the disease. The usefulness of MRI scans is influenced not only by the image quality, the use of adapted MRI presets, and patient preparation protocols but, perhaps most importantly, by the radiologist’s experience in evaluating deep endometriosis. Reports can vary greatly—some are very brief and lack sufficient detail, while others are lengthy, up to three pages of text, and filled with personally selected expressions. The absence of a standardized reporting system complicates the interpretation of MRI reports. A similar situation exists with ultrasound, an even more operator-dependent modality.

It is obvious and essential that in the final step before surgery, these reports are meticulously reviewed and analyzed alongside the recorded images. However, in everyday practice in a standard outpatient endometriosis clinic, where around 20 to 30 patients per day may present with data from different diagnostic centers, a good, concise coding system is invaluable. We check the #ENZIAN score, perform our ultrasound and gynecological exams, and quickly establish an initial diagnosis to plan the next treatment steps. What is crucial and worth underlining is that only one in ten endometriosis patients need to be qualified for surgery. These patients are selected from outpatient admissions, and their image reports are reanalyzed by the multidisciplinary team, often repeated or consulted with other experts. For these patients, we would benefit from additional, facultative #ENZIAN options that could be developed in the #ENZIAN App, likely to be used mainly by advanced tertiary centers. However, for the vast majority who remain on conservative treatment, the #ENZIAN code is entirely sufficient and fulfills its role in our long-lasting care system.

We would like to encourage psychics to try to use #ENZIAN on a regular basis and test its possibilities that show, in our opinion deep understanding of the challenges faced by the community of endometriosis surgeons, even if it is not perfect yet.

In brief:

- In our opinion, the condensed information provided by the #ENZIANu scale (ultrasound), when compared with the #ENZIANm scale (MRI), is more communicative and easier to compare than juxtaposing one-and-a-half-page imaging reports;
- This simplifies both the comparison of imaging methods and the correlation of preoperative and postoperative results, as well as the assessment of the complexity of surgical procedures for individual patients—whether for medical or research purposes.

Given that the topic of a unified and adequate scoring system across medical centers has been seriously debated recently, we reference the findings of the 2024 consensus issued by leading experts in the field of imaging and surgery for patients with endometriosis:

“Non-invasive imaging techniques for the diagnosis of pelvic deep endometriosis and endometriosis classification systems: an International Consensus Statement. Ultrasound Obstet Gynecol. 2024 July”.

Citation: “The majority of participants strongly agreed on the use of TVS or MRI in conjunction with the #Enzian classification, although it is less accurate in cases involving parametrial and USL structures. Future studies on rASRM, AAGL, UBESS, EFI, and the #Enzian classification will hopefully clarify their roles in assessing parametrial and USL involvement”. 

We acknowledge the shortcomings of the scale in assessing the rectum and ureteral regions, as mentioned above. However, of all the available scoring systems, we believe #ENZIAN is the best-suited for surgical purposes and the most promising in further defining advanced aspects of surgery. This direction, in our opinion, should be reflected in the development of the #ENZIAN App, whereas the basic diagram POT ABC should be kept simplified as much as possible to enhance its usability and widespread adoption.

The criteria for various surgical techniques are based on clearly defined qualifications, such as for the bowel—the degree of narrowing, length of infiltration, thickness of infiltration, and distance from the Z-line. In our view, to make the scale fully useful, it should be expanded to include more information for advanced surgeons.

## 5. Limitations

### 5.1. Specificity of the Ward

Due to the fact that the group of patients was not random and originated from only complex cases due to the number of them who needed advanced surgery and were admitted to the specialized oncological gynecology ward, which may correspond with a tertiary endometriosis center, due to its admission criteria, the main limitations of this study might be the following: it is not representative for all endometriosis cases, so it does not show the full application of the #ENZIAN. The system of three-level endometriosis care is under construction in Poland, and our center is nowadays one of the few that can undertake such a level of surgery. 

### 5.2. The Learning Curve

Both TVS and MRI must be considered as improving in efficacy with time, as both the expert surgeon performing the TVSs and the expert radiologist specialized in DE protocol assessment were gaining experience with time, which is obviously an advantage but might have biased the results. 

## 6. Conclusions

3. Preoperative assessment using TVS/TAS + MRI with the #ENZIANi score correlated well with the #ENZIANs postoperative score and demonstrated good concordance in the detection and localization of deep endometriosis, thereby lessening false negative results and enhancing accurate preoperative staging in our tertiary center observation;
4. The ultrasound examination performed by the surgeon achieves the following:

- Enhances the utility of the scale through the direct application of visual representation of the pelvis and concise, practical, repetitive coding;
- Highlights key aspects for the patient, better preparing them for surgery, discussions with the patient, and obtaining full informed consent;
- Improves diagnostic efficiency as the surgeon performing the examination learns to analyze ultrasound images more effectively, which can be compared with images of endometrial changes during surgery. This correlation contributes to better assessment in subsequent examinations;

5. The #ENZIAN classification was invented to meet surgeon needs, and one of its most valuable advantages is its continuous development. Suggestions for future improvements, such as adding the expanded C module or including the ureter and kidney condition, may be considered during the upcoming new edition of “the #ENZIAN app” launch.

## Figures and Tables

**Table 1:** Concordance for #ENZIAN departments.

	k Cohen
P	0.519; SE 0.041 (95% CI, 0.437–0.600)
O left	**0.722**; SE 0.038 (95% CI, 0.648–0.797)
O right	**0.754**; SE 0.036 (95% CI, 0.684–0.823)
T left	**0.795**; SE 0.031 (95% CI, 0.735–0.856)
T right	**0.791**; SE 0.031 (95% CI, 0.729–0.853)
A	**0.690**; SE 0.038 (95% CI, 0.614–0.765)
B left	**0.643**; SE 0.036 (95% CI, 0.573–0.714)
B right	**0.653**; SE 0.036 (95% CI, 0.582–0.723)
C	**0.766**; SE 0.036 (95% CI, 0.695–0.836)
F adenom	**0.776**; SE 0.041 (95% CI, 0.696–0.856)
F bladder	0.448; SE 0.071 (95% CI, 0.309–0.586)
F intest	**0.647**; SE 0.048 (95% CI, 0.553–0.741)
F ureter	0.374; SE 0.050 (95% CI, 0.275–0.472)
F nerve	0.031; SE 0.041 (95% CI, −0.049 to 0.111)
F diaphragm	**0.663**; SE 0.224 (95% CI, 0.224–1.000)
F lung	0.000; SE 0.000 (95% CI, −0.000 to 0.000)
F muscle	−0.033; SE 0.010 (95% CI, −0.053 to −0.013)
F appendix	−0.022; SE 0.009 (95% CI, −0.039 to −0.005)
F bone	−0.011; SE 0.006 (95% CI, −0.023 to 0.001)
F abdominal wall	***0.837***; SE 0.060 (95% CI, 0.720–0.955)

**Bold** denotes a good inter-rater agreement, and ***bold***—a very good inter-rater agreement.

**Table 2:** Results for diagnostic test evaluation.

	Sensitivity, % (95% CI)	Specificity, % (95% CI)	Accuracy, % (95% CI)	PPV, % (95% CI)	NPV, % (95% CI)
P	**98.19 (95.82–99.41)**	83.33 (35.88–99.58)	97.87 (95.43–99.22)	99.63 (97.84–99.94)	50.00 (28.10–71.90)
O left	86.59 (80.40–91.40)	84.16 (75.55–90.67)	85.66 (80.85–89.65)	89.87 (84.94–93.32)	79.44 (72.19–85.19)
O right	87.32 (80.71–92.31)	88.72 (82.08–93.55)	88.00 (83.56–91.59)	89.21 (83.64–93.04)	86.76 (80.91–91.02)
T left	**96.93 (93.78–98.76)**	82.61 (68.58–92.18)	94.53 (91.13–96.90)	96.51 (93.63–98.11)	84.44 (72.12–91.93)
T right	**96.51 (93.23–98.48)**	84.44 (70.54–93.51)	94.53 (91.13–96.90)	96.93 (94.11–98.42)	82.61 (70.39–90.47)
A	**96.31 (93.11–98.30)**	83.33 (67.19–93.63)	94.64 (91.32–96.97)	97.51 (94.96–98.79)	76.92 (63.33–86.55)
B left	**93.55 (89.73–96.27)**	85.29 (68.94–95.05)	92.55 (88.84–95.33)	97.89 (95.38–99.05)	64.44 (52.51–74.81)
B right	**95.31 (91.96–97.55)**	80.77 (60.65–93.45)	93.97 (90.52–96.45)	97.99 (95.69–99.08)	63.64 (49.41–75.82)
C	**95.75 (92.53–97.86)**	76.19 (52.83–91.78)	94.29 (90.89–96.70)	98.02 (95.85–99.07)	59.26 (43.76–73.11)
F adenomyosis	**92.82 (88.25–96.02)**	85.06 (75.80–91.80)	90.43 (86.38–93.60)	93.30 (89.38–95.84)	84.09 (76.00–89.82)
F bladder	48.08 (34.01–62.37)	93.04 (88.95–95.97)	84.75 (80.02–88.74)	60.98 (47.39–73.05)	88.80 (85.89–91.17)
F intestines	88.04 (82.46–92.35)	76.53 (66.89–84.50)	84.04 (79.24–88.12)	87.57 (83.07–91.00)	77.32 (69.41–83.67)
F diaphragm	66.67 (9.43–99.16)	99.64 (98.02–99.99)	99.29 (97.46–99.91)	66.67 (19.46–94.30)	99.64 (98.25–99.93)
F lung	n/a	99.65 (98.04–99.99)	n/a	n/a	100.00 (98.70–100.00)
F muscle	0.00 (0.00–18.53)	97.73 (95.12–99.16)	91.49 (87.60–94.47)	0	93.48 (93.37–93.59)
F appendix	0.00 (0.00–24.71)	98.51 (96.24–99.59)	93.97 (90.52–96.45)	0	95.32 (95.26–95.39)
F bone	0.00 (0.00–40.96)	99.27 (97.40–99.91)	96.81 (94.03–98.53)	0	97.50 (97.48–97.52)
F abdominal wall	83.33 (62.62–95.26)	98.83 (96.63–99.76)	97.51 (94.93–98.99)	86.96 (68.09–95.42)	98.45 (96.29–99.36)

CI, confidence interval; NPV, negative predictive value; PPV, positive predictive value. Additionally, we analyzed off-record the percentage of ureter involvement detected by the renal exam during TAS (Table 3). Numbers in bold correspond to the highest values of sensitivity.

**Table 3:** Results for ureter involvement.

	R (Right Ureter)	R,L (Both Ureters)	L (Left Ureter)
Surgery	19	67	41
Imaging	17	23	25
Imaging%	89.47%	34.33%	60.98%

## Data Availability

The data that support the findings of this study are available on request from the corresponding author.
